# Immune Response following Liver Transplantation Compared to Kidney Transplantation: Usefulness of Monitoring Peripheral Blood CD4+ Adenosine Triphosphate Activity and Cytochrome P450 3A5 Genotype Assay

**DOI:** 10.1155/2013/936063

**Published:** 2013-12-25

**Authors:** Yu Nobuoka, Shugo Mizuno, Kouhei Nishikawa, Kaname Nakatani, Yuichi Muraki, Tomomi Yamada, Masahiro Okuda, Tsutomu Nobori, Yoshiki Sugimura, Shuji Isaji

**Affiliations:** ^1^Department of Hepatobiliary Pancreatic and Transplant Surgery, Mie University School of Medicine, 2-174 Edobashi, Tsu, Mie 514-0001, Japan; ^2^Divisions of Nephro-Urologic Surgery and Andrology, Mie University School of Medicine, 2-174 Edobashi, Tsu, Mie 514-0001, Japan; ^3^Department of Molecular and Laboratory Medicine, Mie University School of Medicine, 2-174 Edobashi, Tsu, Mie 514-0001, Japan; ^4^Department of Pharmacy, Mie University School of Medicine, 2-174 Edobashi, Tsu, Mie 514-0001, Japan; ^5^Translational Medical Science, Mie University School of Medicine, 2-174 Edobashi, Tsu, Mie 514-0001, Japan

## Abstract

Seventy living donor liver transplantation (LDLT) and 39 kidney transplantation (KT) patients were randomly screened by using the peripheral blood CD4+ adenosine triphosphate activity (ATP) assay (IMK assay). The patients were divided into 2 groups in each organ transplantation with low IMK ATP level (<225 ng/mL) or high (>225) (LT-L: *n* = 23, KT-L: *n* = 19, LT-H: *n* = 47, and KT-H: *n* = 20, resp.). The incidence of bacterial and/or viral infection was significantly higher in LT-L group than in LT-H group (74.0 versus 8.5%: *P* < 0.001). Occurrence of total viral infection in KT-L was also significantly higher than that in KT-H (36.8 versus 10%: *P* = 0.046). The sensitivity and specificity of the IMK assay for identifying risk of infection was 0.810 and 0.878 in LDLT patients and 0.727 and 0.607 in KT patients. The percentage of LDLT patients with cytochrome P450 3A5 (CYP3A5) *1/*1 or *1/*3 genotype (expressors) was significantly higher in LT-L group than in LT-H group (53.8 versus 20.7%: *P* = 0.032). In both LDLT and KT patients, the IMK assay can be useful for monitoring immunological aspects of bacterial and/or viral infection. CYP3A5 expressors in LT-L group are related to postoperative infections.

## 1. Introduction

In solid organ transplantation, including liver transplantation (LT) and kidney transplantation (KT), graft and patient survival has been greatly improved during recent two decades, mainly due to the introduction of a variety of immunosuppressive agents including calcineurin inhibitors (CIs) as well as the advances in surgical technique and perioperative management. However, CIs have a narrow therapeutic window, and too little use of immunosuppressive agent may increase the risks of acute and chronic rejection [1], whereas too much immunosuppression may cause infection, malignant disease, and other undesirable adverse effects [2, 3]. The measuring trough levels of CIs combined with laboratory data is widely accepted practice for monitoring solid organ transplants [4, 5], although neither of them is always sensitive or specific for assessing the current immunosuppressive status.

The ImmuKnow (IMK) assay, which was approved by the Food and Drug Administration in 2002, can monitor CD4+ T cell function by measuring the intracellular concentration of adenosine triphosphate (ATP). This assay has been used for identifying transplant patients at risk for infection (with low IMK ATP levels) or rejection (with high IMK ATP levels) [6, 7], whereas others argue against its predictive usefulness [8, 9]. In each organ transplant recipient, the true benefit of IMK assay for monitoring of immunological aspects needs to be clarified.

In LT and KT patients, most widely used immunosuppressive drug is tacrolimus, which is mainly metabolized by cytochrome P450 (CYP) 3A4 and CYP3A5 in the small intestine and the liver [10, 11]. In other words, in LT but not KT patients, the CYP3 genotypes of recipients as well as donors affect the blood concentration of tacrolimus. LT often requires less immunosuppression compared to KT and other solid organ transplantations, and one of the reasons might be metabolism of tacrolimus in LT patients. Although CYP3A5 plays a key role in the pharmacokinetics of tacrolimus especially in living donor LT (LDLT) patients [12] as well as KT patients [13, 14], the influence of CYP3A5 genotype on immune function following transplantation still remains unclear.

We hypothesized that the IMK assay can be useful for monitoring of immunological aspects in LT as well as KT patients and that the CYP3A5 genotypes also affect postoperative immune functions following KT in addition to LT. The aim of this study is to evaluate the immune reaction after LT comparing KT by using the IMK assay and CYP3A5 genotype.

## 2. Patients and Methods

### 2.1. Patients

Ninety-eight LDLT (March 2002 to December 2012) and 39 KT (October 1980 to December 2012) patients, who underwent operation and had been followed as an outpatient at the Mie University Hospital, were candidates for this study. The inclusion criteria were LDLT and KT patients, who happened to be treated either as inpatients or who had return visits to the clinic during the period of January 2010 to December 2012. The only exclusion criterion was if the patient was followed at another center than the Mie University Hospital. This study was retrospective cohort study. A total of 70 LDLT patients and 39 KT patients were screened using the IMK assay and observed clinically. According to the previous report [6] that 225 ng/mL was the cutoff ATP level for identifying risk of infection, we classified these patients into four groups as follows: LT-L group: LDLT patients in whom at least 1 IMK ATP level was <225 ng/mL, LT-H group: LDLT patients in whom no IMK ATP level was <225 ng/mL, KT-L group: KT patients in whom at least 1 IMK ATP level was <225 ng/mL, and KT-H group: KT patients in whom no IMK ATP level was <225 ng/mL. The complete medical records of each patient were obtained. Infection was defined as a patient who required antibiotics and/or antiviral agents. Acute cellular rejection was defined by the 9-point Banff rejection activity index [15] as mild, moderate, or severe based on a liver biopsy at the time of undergoing the IMK assay when rejection was suspected clinically. The CYP3A5 genotypes were examined in 42 LT and 23 KT patients who underwent transplantation after September 2005 [16]. This study (IMK assay and CYP3A5 genotypes) was approved by institutional review board at Mie University Hospital and each patient's consent was obtained.

### 2.2. ImmuKnow (IMK) Assay

Blood samples were collected in sodium heparin tubes, and the intracellular adenosine triphosphate activity (ATP) level was measured by using IMK assay kit (MBL, Nagoya, Japan). Blood samples were processed on the day of sample collection. Briefly, 250 *μ*L of anticoagulated whole blood was diluted with the provided sample diluent to make a final volume of 1000 *μ*L. Samples were added to wells of a 96-well plate and incubated for 15 to 18 h with phytohemagglutinin at 37°C and 5% CO_2_ atmosphere. After enrichment of CD4+ T cells by addition of magnetic particles coated with an anti-human CD4 monoclonal antibody (Dynabeads, Dynal, Oslo, Norway), cells were washed and lysed to release intracellular ATP. Released ATP was measured with a luciferin/luciferase assay in a luminometer. The patient's level of immune response was expressed as the amount of ATP (ng/mL). According to the previous report [6], we used the cutoff ATP level of 225 ng/mL for identifying risk of infection and 525 ng/mL for rejection, and we defined a target immunological response zone ranging 226–525 ng/mL.

### 2.3. Immunosuppression

In LT patients, the immunosuppression protocol consisted of tacrolimus and low-dose steroids. The target blood trough level for tacrolimus was 10 to 12 ng/mL during the first 2 weeks, approximately 10 ng/mL thereafter, and 5 to 10 ng/mL from the second month after LDLT. If their liver function was stable, recipients were weaned off steroids at 3 months after LDLT. In KT patients, twenty-nine (74.4%) patients used tacrolimus and 8 (20.5%) used cyclosporine. The target blood trough level for tacrolimus was around 5 ng/mL. Two (5.1%) patients did not use a calcineurin inhibitor. Twenty-nine (74.4%) patients received mycophenolate mofetil.

### 2.4. Evaluation of Tacrolimus Blood Concentration and Concentration/Dose (*C*/*D*) Ratio

The tacrolimus blood concentration was then measured by using a semiautomated microparticle enzyme immunoassay (IMx, Abbott Co., Ltd., Tokyo, Japan). The daily dose of tacrolimus was recorded and its weight-adjusted dosage (mg/kg per day) was calculated. Then, the measured blood tacrolimus concentration was normalized by the corresponding dose per body weight 24 h before blood sampling to obtain the concentration/dose (*C*/*D*) ratio, which was then used for estimating the tacrolimus dose needed to achieve the target trough concentration.

### 2.5. Genotyping of Cytochrome P450 3A5

According to our previous report [16], the CYP3A5 A6986G (rs776746) polymorphism was analyzed for the detection of the *3 allele, because CYP3A5*3 is the major defective allele and because of the fact that other functional exonic SNPs are rare in the Japanese population [17]. With regard to the CYP3A5 genotype, patients were allocated into 2 groups: CYP3A5*1/*1 or CYP3A5*1/*3 (expressors) and CYP3A5*3/*3 (nonexpressors).

### 2.6. Definitions of Cytomegalo Virus (CMV) and Bacterial Infection

CMV infection was defined as a febrile illness in the presence of clinical symptoms and the detection of CMV in the blood (by quantitative nucleic acid testing or antigenemia). When the patient was positive for pp65 antigenemia or had more than 10,000 CMV copies/*μ*L in the blood, we administered a 2- to 4-week course of intravenous ganciclovir followed by oral valganciclovir after hospital discharge. Bacterial infection was defined as a febrile illness with clinical symptoms and the detection of bacteria in sputum, abdominal fluid, or blood. When the patient had a bacterial infection, antibiotics against susceptible bacteria were administered until the clinical symptoms improved.

### 2.7. Statistical Analyses

All values were expressed as the mean ± standard deviation (SD) and median as appropriate. Pearson's correlation coefficient was used to determine the relationship between the blood concentration of tacrolimus and the dosage of tacrolimus and between the blood concentration of tacrolimus and the IMK ATP levels in LT and KT patients. Fisher's exact tests were used for categorical factors. Student's *t*-test was used to compare ATP levels and tacrolimus *C*/*D* ratio between LT-L and LT-H, KT-L and KT-H. The data were analyzed using statistics computer software Pharmaco Analyst II (Hakuhousha Co., Tokyo, Japan). A *P* value <0.05 was considered to indicate a statistically significant difference.

## 3. Results

Patients' characteristics of LDLT patients were shown in Table 1. There were 23 patients in LT-L group and 47 in LT-H group. The two groups were similar in age, male/female ratio, the model for end-stage liver disease (MELD) score, and etiology of LDLT. There were also no differences between the two groups in CMV serology tests in each donor and recipient. The CYP3A5 genotypes were examined in 42 patients (LT-L group: 13 and LT-H group: 29) who underwent LDLT after September 2005. In LDLT recipients, the percentage of them with *1/*1 or *1/*3 genotype (expressors) was significantly higher in LT-L group than in LT-H group (53.8% versus 20.7%, *P* = 0.032). In LDLT donors, there was no significant difference between the 2 groups. There was no significant difference in median interval time after LDLT.

In KT patients, 19 patients were in KT-L group and 20 were in KT-H group (Table 2). There was no significant difference in age, male/female ratio, type of KT, ratio of ABO-incompatible, ratio of tacrolimus use, and CMV serology tests in each donor and recipient. The CYP3A5 genotypes were examined in 23 KT patients (KT-L group: 11 and KT-H group: 12); there was no significant difference between the 2 groups. There was also no significant difference in median interval time after LDLT.

### 3.1. Pharmacokinetics of Tacrolimus and IMK ATP Level

**Figure 1:**
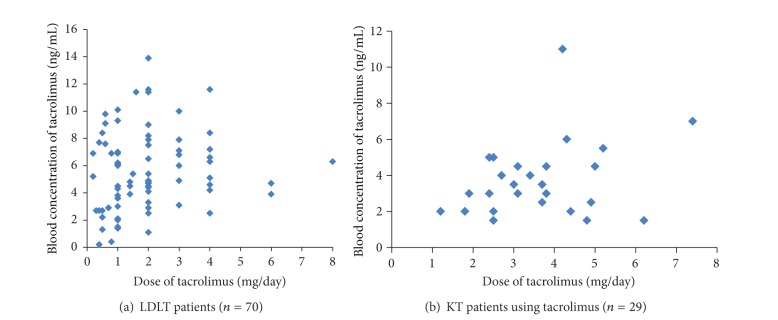
Relationship between the blood concentration of tacrolimus and dose of tacrolimus. There was no statistically significant relationship between the blood concentrations of tacrolimus and the dosage of tacrolimus in LDLT recipients (*R* = 0.154, *P* = 0.158) (a) and in KT patients who used tacrolimus (*R* = 0.292, *P* = 0.1162) (b).

**Figure 2:**
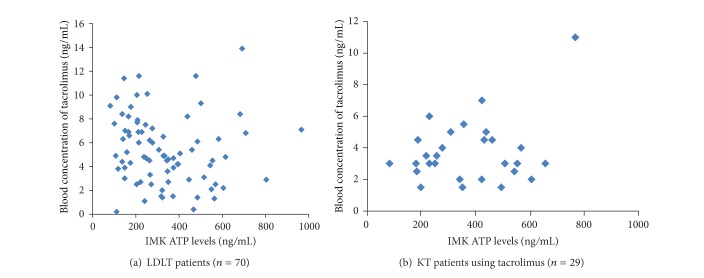
Relationship between blood concentration of tacrolimus and IMK ATP levels. There was also no statistically significant relationship between the blood concentrations of tacrolimus and the IMK ATP levels in LDLT recipients (*R* = 0.147, *P* = 0.181) (a) and in KT patients who used tacrolimus (*R* = 0.284, *P* = 0.2745) (b).

There was no statistically significant relationship between the blood concentrations of tacrolimus and the dosage of tacrolimus in LDLT recipients (*R* = 0.154, *P* = 0.158) (Figure 1(a)) and in KT patients who used tacrolimus (*R* = 0.292, *P* = 0.1162) (Figure 1(b)). There was also no statistically significant relationship between the blood concentrations of tacrolimus and the IMK ATP levels in LDLT recipients (*R* = 0.147, *P* = 0.181) (Figure 2(a)) and in KT patients who used tacrolimus (*R* = 0.284, *P* = 0.2745) (Figure 2(b)). Clinically, there were no samples that behave like outliers in Figures 1 and 2.

### 3.2. IMK ATP Level and Tacrolimus *C*/*D* Ratio in LT and KT Patients

**Figure 3:**
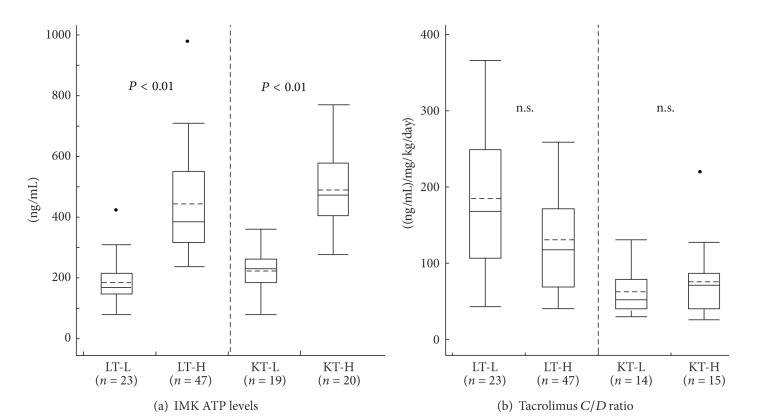
IMK ATP levels and tacrolimus *C*/*D* ratio in LT and KT patients. (a) The mean ATP levels in LT-L patients was significantly lower than that in LT-H (185.7 (82–310) ng/mL versus 442.7 (238–966), *P* < 0.01), and KT-L patients had also lower IMK ATP levels than KT-H patients (225.6 (80–359) ng/mL versus 488.6 (277–770), *P* < 0.01). (b) The mean tacrolimus *C*/*D* ratios were 184.5 (43–366) ng/mL per mg/kg/day in LT-L patients and 130.5 (41–460) in LT-H without any significant difference *P* = 0.091. There was also no significant difference between KT-L and KT-H (62.2 (30–131) ng/mL per mg/kg/day versus 76.0 (26–248), *P* = 0.440).

The mean ATP levels in LT-L patients were significantly lower than those in LT-H (185.7 (82–310) ng/mL versus 442.7 (238–966), *P* < 0.01), and KT-L patients had also lower IMK ATP levels than KT-H patients (225.6 (80–359) ng/mL versus 488.6 (277–770), *P* < 0.01) (Figure 3(a)). The mean tacrolimus *C*/*D* ratios were 184.5 (43–366) ng/mL per mg/kg/day in LT-L patients and 130.5 (41–460) in LT-H without any significant difference *P* = 0.091. There was also no significant difference between KT-L and KT-H (62.2 (30–131) ng/mL per mg/kg/day versus 76.0 (26–248), *P* = 0.440) (Figure 3(a)).

### 3.3. Occurrence of Rejection and Infection in LT and KT Patients

**Figure 4:**
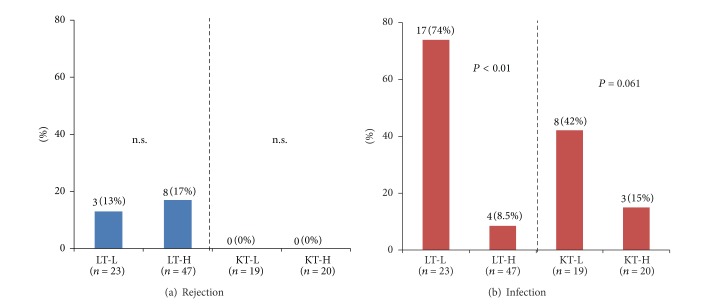
Occurrence of rejection and infection in LT and KT patients. (a) Histologically proven rejection occurred in 3 cases (13%) in LT-L group and in 8 cases (17%) in LT-H group during this survey period. There was no significant difference between the two groups (*P* = 0.668). No rejection occurred in both KT-L and KT-H patients. (b) The incidence of bacterial and/or viral infection was significantly higher in LT-L group (74%) than in LT-H group (8.5%) (*P* < 0.001). Posttransplant infection occurred in 8 patients (42%) in KT-L group compared to 3 patients (15%) in KT-H group (*P* = 0.061).

Histologically proven rejection occurred in 3 cases (13%) in LT-L group and in 8 cases (17%) in LT-H group during this survey period. There was no significant difference between the two groups (*P* = 0.668). No rejection occurred in both KT-L and KT-H patients (Figure 4(a)). The incidence of bacterial and/or viral infection was significantly higher in LT-L group (74%) than in LT-H group (8.5%) (*P* < 0.001). Posttransplant infection occurred in 8 patients (42%) in KT-L group compared to 3 patients (15%) in KT-H group (*P* = 0.061) (Figure 4(b)).

### 3.4. Occurrence of Infection in LT and KT Patients according to IMK ATP Levels

During this survey, 20 LDLT patients experienced bacterial infection and 17 suffered from viral infection including CMV infection and recurrence of hepatitis C (HCV) (Table 3). In LDLT patients, occurrences of all kinds of infections in LT-L were significantly higher than those in LT-H (*P* < 0.01). In KT patients, 2 experienced bacterial infection and 9 suffered from viral infections. Occurrence of total viral infection in KT-L was significantly higher than that in KT-H (*P* = 0.046).

When we used cutoff ATP level of 225 ng/mL for identifying risk of infection according to the previous report [6], diagnostic accuracy of IMK was favorable with sensitivity of 0.810 and specificity of 0.878 in LDLT patients and was also satisfactory with sensitivity of 0.727 and specificity of 0.607 in KT patients.

## 4. Discussion

Infectious diseases for all types of transplant patients remain important and sometimes serious complications mainly due to monitoring immunosuppressive status [18]. In addition to trough levels of CIs combined with laboratory data, the IMK assay is a potentially useful tool for predicting the development of infections and rejection episodes in LT recipients [19]. On the other hand, another meta-analysis [20] suggested that IMK is not able to identify individuals at risk of infection or rejection and additional studies are still needed to clarify the usefulness of this test, though there is a significant heterogeneity between studies including different allograft types, control groups, and cutoff values. In the current study, we evaluated IMK assay for both LT and KT patients at the same institution by using the same cutoff value (the IMK ATP level: 225 ng/mL), because there might be differences in posttransplant immune reaction between LT and KT, and we focused on the differences in each organ transplant.

In our study, we had 23 (32.9%) LDLT patients with low immune reaction (LT-L) and most of them had experienced bacterial and/or viral infections, though there was no difference in tacrolimus *C*/*D* ratio between LT-L and LT-H. The diagnostic accuracy of IMK assay for identifying risk of infection was also favorable in LDLT patients. These results suggested that the IMK assay was a useful diagnostic tool for identifying risk of infection in LDLT patients, as previous report [19]. Regarding the LDLT patients with HCV, which is specific pathogenesis of the graft liver after LDLT, 5 out of 6 recurrent HCV cases were belonging to LT-L group. The IMK assay also can be additional valuable tool to discriminate recurrent HCV and acute rejection.

In KT patients, there were 19 (48.7%) recipients with low immune reaction (KT-L) and 8 out of them (42%) had experienced bacterial and/or viral infections with no significant difference between KT-L and KT-H except for total viral infection cases. The sensitivity and specificity of the IMK assay for identifying risk of infection were also satisfactory in KT patients, but lower than those in LDLT patients. In KT but not LT patients, the true benefit of IMK assay for monitoring of immunological aspects is still controversial. Although several studies suggested that the IMK assay may be a potentially useful predictor for the adverse events after KT [6, 7], Huskey et al. [8] reported that single time point IMK assay does not aid in the prediction of future opportunistic infections by using the same cutoff value (225 ng/mL) as our study. The IMK levels in KT patients were relatively low comparing to LT patients, and the cutoff level for identifying risk of infection might be better to define as lower than 225 ng/mL.

When we focused on the occurrence of infection in LT and KT patients according to IMK ATP levels, LT patients tend to suffer from infection though the IMK ATP levels in LT patients were relatively high comparing to KT patients. This might be because the liver plays a role of metabolism of immunosuppressive drugs, besides the difference in surgical stress between LT and KT.

CYP3A5 is known to play a key role in the pharmacokinetics of tacrolimus in LT [12] and KT patients [13, 14], and intestinal CYP3A5 as well as hepatic CYP3A5 plays an important role in the first-pass effect of orally administered tacrolimus until 35 days [21]. In KT patients, the liver and intestinal functions are usually normal even during perioperative period, on the other hand, LT patients sometimes encounter graft liver and/or intestinal dysfunction due to postoperative complications including rejection, portal hypertension, or vascular complications, especially on early phase after LT.

We previously reported that the tacrolimus *C*/*D* ratio was significantly lower in subjects with CYP3A5*1 alleles (expressor) than in those with the CYP3A5*3 allele (nonexpressor), suggesting that high dose of tacrolimus is required in expressors to achieve the target trough levels [16]. In LT patients of our study, the percentage of CYP3A5 expressors as well as the occurrence of infections was significantly higher in LT-L group than in LT-H group, which means that LT-L patients with expressors tend to be over immunosuppressive because of achieving the target trough levels, resulting in infections. However, in KT patients, there was no association between CYP3A5 and infectious complication despite the small number. One of the reasons of this result might be inadequate cutoff value of the IMK ATP levels but still remains unknown.

Our study has some limitations including the small sample size, its retrospective nature, and the use of single time point measurements of the IMK ATP levels in some patients. The IMK assays were undergone at sometimes haphazard times and no clear reasons for this assay could be identified, which means either inadequate ancillary studies or questionable indications for the IMK assays. None of allograft rejection episodes in KT rendered the analysis of this subgroup very difficult. In addition, the CYP3A5 genotypes were examined in only 42 LDLT patients (60%) and 23 KT patients (59%).

## 5. Conclusion

In conclusion, our study identifies that the IMK assay can be useful for monitoring immunological aspects of bacterial and/or viral infection for both LT and KT patients. CYP3A5 expressors, affecting tacrolimus pharmacokinetics, in LT-L group are related to postoperative infections.

## Figures and Tables

**Table 1:** Backgrounds of LDLT patients.

	LT-L group (*n* = 23)	LT-H group (*n* = 47)	*P* value
	IMK < 225		
Average age	43.6 (3–65)	41.7 (1–68)	NS
Male/female	16/7	32/15	NS
MELD score	14.1 (9–32)	16.0 (8–35)	NS
Etiology of LDLT			
HCV (HCC)	8 (5)	15 (8)	NS
HBV (HCC)	2 (0)	4 (2)	
PBC	3	10	
BA	1	6	
Alcohol	4 (2)	4	
Others	5	8	
CMV serology test			
R-seropositive	5 (21.7%)	12 (25.5%)	NS
D-positive/R-negative	8 (37.8%)	15 (31.9%)	
D-negative/R-negative	10 (43.5%)	20 (42.5%)	
R-CYP3A5			
*1*1 or *1*3	7 (53.8%)	6 (20.7%)	0.032
*3*3	6	23	
D-CYP3A5			
*1*1 or *1*3	6 (46.2%)	13 (44.8%)	NS
*3*3	7	16	
Median months after LDLT	39.2 (1.4–71.4)	43.1 (1.8–67.5)	NS

IMK: ImmuKnow, MELD: model for endstage liver disease, LDLT: living donor liver transplantation, HCV: hepatitis C, HCC: hepatocellular carcinoma, HBV: hepatitis B, PBC: primary biliary cirrhosis, BA: biliary atresia, CYP3A5: cytochrome P450 3A5, CMV: cytomegalovirus, R-: recipient, and D-: donor.

**Table 2:** Backgrounds of KT patients.

	Group KT-L (*n* = 19)	Group KT-H (*n* = 20)	*P* value
	MK < 225		
Average age	48.8 (35–75)	48.9 (22–68)	NS
Male/female	12/7	15/5	NS
Type of KT			
Living donor KT	2 (10.5%)	7 (35%)	NS
Deceased donor KT	17	13	
ABO-incompatible	4 (21.1%)	3 (30%)	NS
Tacrolimus use	14 (73.7%)	15 (75%)	NS
CMV serology test			
R-seropositive	11 (68.7%)	10 (66.7%)	NS
D-positive/R-negative	5	5	
R-CYP3A5			
*1*1 or *1*3	5 (45.5%)	3 (33.3%)	NS
*3*3	6	9	
Median months after KT	19.1 (3.5–385)	22.4 (1.2–322)	NS

KT: kidney transplantation, IMK: ImmuKnow, CMV: cytomegalovirus, CYP3A5: cytochrome P450 3A5, R-: recipient, and D-: donor.

**Table 3:** Occurrence of infection in LT and KT patients according to IMK ATP levels.

Infection	Group LT-L (*n* = 23)	Group LT-H (*n* = 47)	*P* value
	IMK < 225	IMK > 225	
Bacterial infection	17 (73.9%)	3 (6.4%)	<0.01
Viral infection	15 (65.2%)	2 (4.2%)	<0.01
CMV	10 (43.5%)	1 (2.1%)	<0.01
HCV	5/8 (62.5%)	1/15 (6.7%)	<0.01

Infection	Group KT-L (*n* = 19)	Group KT-H (*n* = 20)	*P* value
	IMK < 225	IMK > 225	

Bacterial infection	1 (5.3%)	1 (5%)	n.s.
Viral infection	7 (36.8%)	2 (10%)	0.046
BKV	1 (5.3%)	2 (10%)	n.s.
CMV	3 (15.8%)	0	n.s.
VZV	2 (10.5%)	0	n.s.
HSV	1 (5.3%)	0	n.s.

CMV: cytomegalovirus, HCV: recurrence of hepatitis C infection, BKV: BK virus, VZV: varicella-zoster virus, HSV: herpes simplex virus, and n.s.: not significant.

## List of Abbreviations

IMK: ImmuKnow immune cell function assay
CYP3A5: Cytochrome P450 3A5
LDLT: Living donor liver transplantation
LT: Liver transplantation
KT: Kidney transplantation
*C*/*D* ratio: Concentration/dose ratio.
